# Factors associated with suicide in people who use drugs: a scoping review

**DOI:** 10.1186/s12888-023-05131-x

**Published:** 2023-09-05

**Authors:** Joan Devin, Suzi Lyons, Lisa Murphy, Michael O’Sullivan, Ena Lynn

**Affiliations:** 1 Health Research Board, Grattan House, 67–72 Lower Mount Street, Dublin 2, Ireland; 2 School of Pharmacy and Biomolecular Sciences, Royal College of Surgeons in Ireland, 1st Floor Ardilaun House Block B, 111 St Stephen’s Green, Dublin 2, Ireland

**Keywords:** Suicide, Drug use, Risk factors, Opioids, Sex

## Abstract

**Background:**

Suicide is a significant contributor to global mortality. People who use drugs (PWUD) are at increased risk of death by suicide relative to the general population, but there is a lack of information on associated candidate factors for suicide in this group. The aim of this study was to provide a comprehensive overview of existing evidence on potential factors for death by suicide in PWUD.

**Methods:**

A scoping review was conducted according to the Arksey and O’Malley framework. Articles were identified using Medline, CINAHL, PsycINFO, SOCIndex, the Cochrane Database of Systematic Reviews and the Campbell Collaboration Database of Systematic Reviews; supplemented by grey literature, technical reports, and consultation with experts. No limitations were placed on study design. Publications in English from January 2000 to December 2021 were included. Two reviewers independently screened full-text publications for inclusion. Extracted data were collated using tables and accompanying narrative descriptive summaries. The review was reported using the Preferred Reporting Items for Systematic Reviews and Meta-Analyses Extension for Scoping Reviews (PRISMA-ScR) guidelines.

**Results:**

The initial search identified 12,389 individual publications, of which 53 met the inclusion criteria. The majority (87%) of included publications were primary research, with an uncontrolled, retrospective study design. The most common data sources were drug treatment databases or national death indexes. Eleven potential factors associated with death by suicide among PWUD were identified: sex; mental health conditions; periods of heightened vulnerability; age profile; use of stimulants, cannabis, or new psychoactive substances; specific medical conditions; lack of dual diagnosis service provision; homelessness; incarceration; intravenous drug use; and race or ethnicity. Opioids, followed by cannabis and stimulant drugs were the most prevalent drugs of use in PWUD who died by suicide. A large proportion of evidence was related to opioid use; therefore, more primary research on suicide and explicit risk factors is required.

**Conclusions:**

The majority of studies exploring factors associated with death by suicide among PWUD involved descriptive epidemiological data, with limited in-depth analyses of explicit risk factors. To prevent suicide in PWUD, it is important to consider potential risk factors and type of drug use, and to tailor policies and practices accordingly.

**Supplementary Information:**

The online version contains supplementary material available at 10.1186/s12888-023-05131-x.

## Background

Suicide is a significant global public health concern [1, 2]. The World Health Organization (WHO) estimates that over 700,000 people die by suicide each year, with more deaths attributed to suicide than malaria, HIV/AIDS, breast cancer, or war and homicide [3]. The Global Burden of Disease Study 2016 [2] found that while age standardised mortality rates for suicide have greatly reduced since 1990, suicide remains an important contributor to mortality.

Suicide is defined as a death caused by intentional, self-directed injury [4]. The factors that contribute to suicide are complex and wide-ranging [1, 5, 6]. Suicidal behaviour varies according to sex, age, geographic distribution, and socio-political setting [3, 7, 8]. Rates of suicide are consistently higher in men than in women, although women outnumber men in suicide attempts [3, 7, 9, 10].

The effects of suicide in society are significant. For the families, friends and communities bereaved through suicide there is a severe emotional toll [11–13]. Direct monetary costs linked to suicide include the cost of emergency services, medical care, medicolegal costs and funeral expenses, while indirect costs to society include loss of earnings due to premature mortality [14, 15]. The WHO Comprehensive Mental Health Action Plan 2013–2030 [16] sets a target of reducing global suicide mortality by one third by 2030. A defined action for WHO Member States to reach this target, is the development and implementation of strategies for mental health promotion and suicide prevention, with emphasis on locally-identified vulnerable and marginalized groups, with a recommendation to include people with mental disorders as a vulnerable and marginalized group [16].

One such vulnerable population known to be at increased risk of death by suicide are people who use drugs (PWUD) [17–21]. Evidence from epidemiological and clinical research indicates a 7- to 22-fold increase in suicide mortality among PWUD relative to that expected in the general population [20–23]. While there have been several literature reviews on risk factors for suicide among PWUD [5, 19, 24–26], and previous systematic reviews [18, 23, 27–29] and meta-analyses [22, 30, 31] that aimed to quantify the association of problem drug use with suicide mortality among high-risk groups, no study has sought to systematically identify and thematically map the available evidence on potential factors associated with suicide among PWUD.

Suicide prevention strategies may be universal (such as mental health policies, alcohol reduction policies, and restricting access to means of suicide), or targeted and selective (such as strategies focusing on young people, or education programmes for doctors to help them identify at-risk individuals) [1, 16, 32]. Given that PWUD remain a high-risk group for dying by suicide, they may not benefit from universal prevention strategies to the same extent as the general population. Therefore, understanding specific, characteristics, risks and the contexts in which risk may be amplified in this population are critical precursors to developing targeted interventions and suicide prevention strategies. Due to the limited clarity on the extent, range, and nature of the evidence regarding factors associated with death by suicide among PWUD, as well as ambiguity regarding the overall progress and direction of this field of research, a scoping review was judged to be an appropriate study design to address this issue.

## Objectives

The aim of this review was to provide a comprehensive overview of existing evidence on factors associated with death by suicide, specifically among PWUD, using a scoping review methodology. The objectives were:

- To map the extent, range, and nature of available evidence on factors associated with death by suicide among PWUD.
- To identify knowledge gaps and limitations in this body of evidence, and.
- To inform suicide prevention policy and best practice guidelines for working with PWUD, where appropriate.

## Methods

Scoping reviews are an increasingly popular form of knowledge synthesis that aim to systematically search and map the breadth of available evidence (including evidence in published and grey literature), categorise key concepts, identify knowledge gaps and research deficits, and propose recommendations to guide future research [33, 34]. A key characteristic of a scoping review is the incorporation of stakeholder consultation into the methodological framework to both inform and validate the study findings [35]. This process provides opportunity for knowledge transfer and exchange with experts working at the intersection of research, policy and practice.

The review was guided by the methodological framework for scoping reviews outlined by Arksey and O’Malley [36], and updated by Peters et al. [35]. This framework involves six stages, discussed in further detail below. The scoping review was reported in accordance with the Preferred Reporting Items for Systematic Reviews and Meta-Analyses Extension for Scoping Reviews (PRISMA-ScR) (Appendix 1) [37]. A protocol for this study was previously published in 2021 [38].

### Stage 1: Identifying the research question

The following research question was identified based on the overarching aim of the scoping review: What is the extent, range, and nature of evidence regarding factors associated with death by suicide among PWUD? Further explanation of the definitions used to guide the research question are provided in Appendix 2.

### Stage 2: Identify and retrieve relevant items

A comprehensive search strategy to identify relevant literature was developed in accordance with scoping review guidance and was peer-reviewed by an information specialist [39].

The inclusion and exclusion criteria for the review were developed through an iterative process as the searches progressed. For the purposes of the scoping review, PWUD was considered an umbrella phrase under which various terms indicative of problem drug use are subsumed, including, but not limited to, any of the following: people who use, misuse, or abuse drugs (including non-medical use of licit drugs and illicit drug use); people with a diagnosis of substance use disorder (SUD) / drug use disorder (DUD); people with drug dependence; people who are regular or ‘casual’ users of drugs; and people who report recent drug use. All peer-reviewed and non-peer-reviewed articles, reports, and reviews published in the English language were eligible for inclusion. Searches were limited to evidence sources published between January 2000 and December 2021 inclusive, with the most recent literature search executed in December 2021. No limitations were placed on study design. Full inclusion and exclusion criteria are provided in Table 1.

The bibliographic databases Medline, CINAHL, PsycINFO, SOCIndex, the Cochrane Database of Systematic Reviews, and the Campbell Collaboration Database of Systematic Reviews were searched. Key academic journals were hand searched for relevant published articles. Grey literature databases, including Open Grey, were searched using keywords and phrases identified in published literature. Finally, the review team contacted academic experts, professional societies and relevant organisations to ascertain the availability of any additional evidence sources not identified in previous searches. See Appendix 3 for full search terms.

**Table 1:** Inclusion and exclusion criteria for study selection

Included	Excluded
*Population: People who use drugs (PWUD)*	
• Sources in which it is made explicit that the participant group (or a subgroup) were PWUD	• Sources in which is it not made explicit that the deceased (or a subgroup) were PWUD
• Sources that include a participant group (or subgroup) who use, abuse or are dependent on drugs *only* OR • Sources that include a participant group (or subgroup) who use, abuse or are dependent on *both* drugs and alcohol	• Sources that include a participant group (or subgroup) who use, abuse or are dependent on alcohol *only* OR • Sources in which the participant group (or a subgroup) is only identified as having substance use problems, which could be related to alcohol alone, drug(s) alone or a combination of both
• Sources involving adult participants or participants in late adolescence	• Sources involving children or early adolescents (below 15) *only*
*Concept: Factors*	
• Sources that explicitly identify a variable, or several variables, as factors associated with death by suicide among PWUD OR • Sources in which factors can be inferred (e.g. sources that report sex segregated data)	• Sources that do not explicitly analyse factors associated with death by suicide among PWUD OR • Sources in which factors cannot be inferred
*Outcome: Suicide*	
• Sources in which the primary outcome variable (or one of several outcome variables) is death by suicide	• Sources that focus on suicide ideation, non-fatal attempted suicide, non-fatal deliberate self-harm, or accidental overdose *only* OR • Sources that focus on all-cause mortality among PWUD *only* OR • Sources that focus on the means of suicide death, such as drug concentrations, *only*
• Sources that include death via overdose (or poisoning) as an outcome and analyse deliberate overdose (or poisoning) deaths as a distinct subgroup	• Sources in which overdose (or poisoning) is a primary outcome but intentionality is not made explicit (i.e. no differentiation between intentional or accidental overdose deaths)
*Context*	
• Sources that provide insight into risk factors for death by suicide among PWUD across all settings, including before, during and after drug treatment, psychiatric treatment and incarceration, and other legal or social care contexts	• Sources in which the illicit use of a drug or drugs was solely to complete suicide (i.e., intentional injecting of insulin, which was not prescribed to the individual, for the purpose of completing suicide)
• Sources from any geographic region	• Sources without English language full text

### Stage 3: Selecting studies

Titles and abstracts retrieved from databases were screened in Eppi-Reviewer 4.0, a software program for managing and analysing data used in literature reviews, including scoping reviews [40]. Four reviewers (EL, LM, MOS, and SL) screened all titles and abstracts against the inclusion criteria. At the full-text stage of screening, pdf copies of the relevant publications were imported to and managed using the Zotero bibliographic management software and reviewed independently by two reviewers (JD and EL). Reasons for exclusion of full texts included: study not focused on PWUD, suicide deaths not an outcome, studies with a pathology or toxicology focus, use of illicit drugs only to complete the act of suicide, or if clear factors for death by suicide could not be identified. Quality appraisal of full texts was not performed, as this is generally not recommended in scoping reviews because the aim is to map the available evidence rather than provide a synthesized and clinically meaningful answer to a question [39]. Where the review team identified sources with obvious overlap in either participant samples or datasets, sources that provide the most information relevant to the aims of the scoping review only were included. Any uncertainty in relation to publication eligibility was resolved through discussion with the other authors.

### Stage 4: Mapping/charting the data

**Fig. 1:**
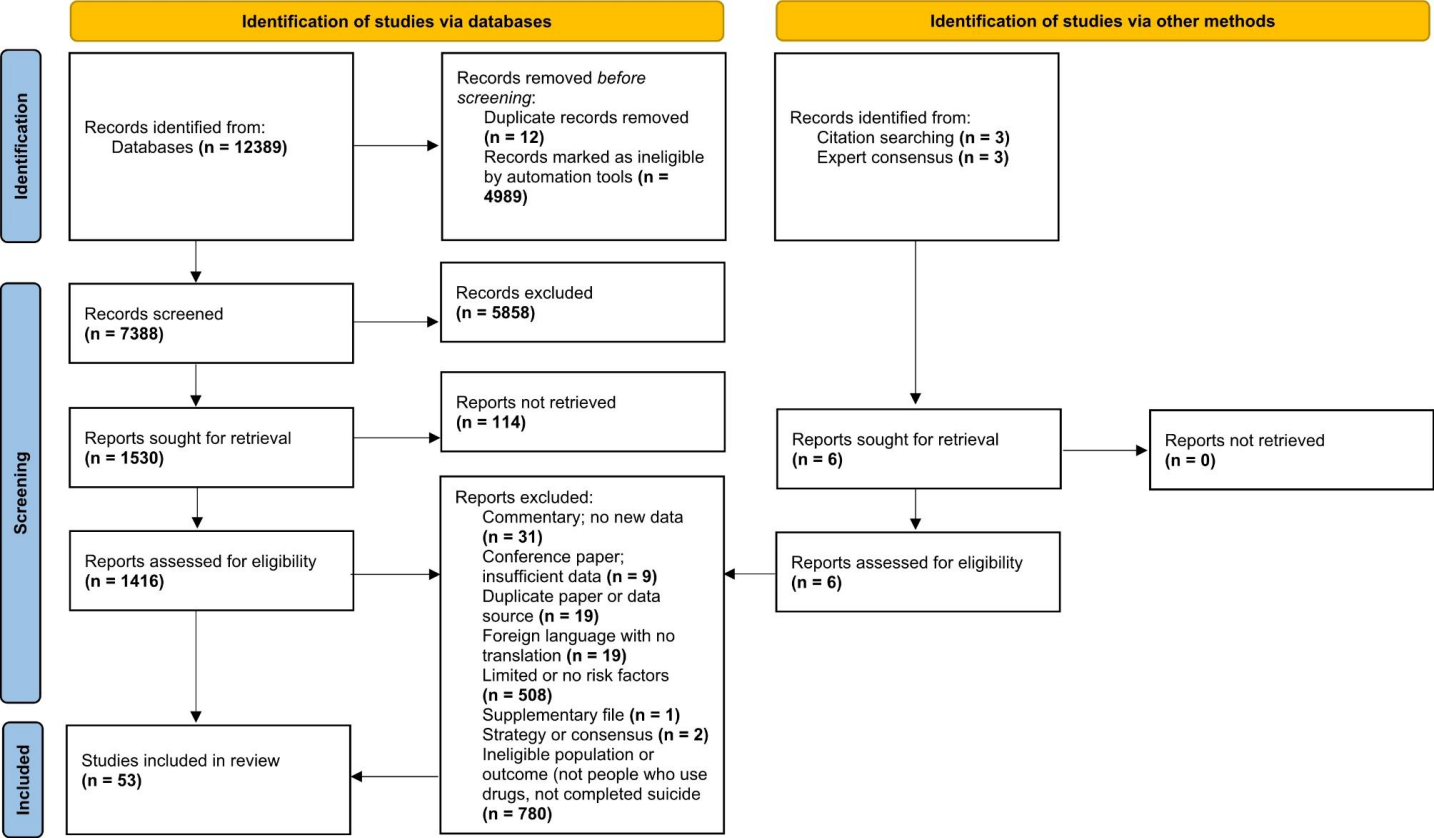
PRISMA (Flow diagram of study selection [41]

Full texts were examined and sorted in Zotero according to emerging themes. Data were charted by all authors using a data charting form. Data identified for extraction were informed by the purpose of the scoping review and, as with the other stages, this was an iterative process, progressing as the charting of this scoping review developed. Consultation took place throughout the data charting process with literature excluded if the authors agreed through consensus that there was insufficient data on the topic. The following information was collated on the data charting form: study characteristics, aim of the study/report, study design, setting, population characteristics, the use of diagnostic inclusion criteria for drug use or the authors definition of drug use, the presence/absence of a control or comparison group, definition of suicide, risk factors for death by suicide, data analysis, the main findings, interpretation of findings, recommendations for future research, study limitations, and themes. A final selection of 53 studies was agreed for inclusion. This included three additional publications captured through the consultation exercise (Fig. 1).

### Stage 5: Collating, summarising, and reporting the results

The data were collated and summarised in accordance with the overall aim and objectives of the scoping review. A narrative account of the findings was presented. Descriptive analysis of studies included information related to geographic distribution, publication dates, evidence source, study design, and primary drug of focus. An overview of research limitations, and considerations for policy and practice extracted from reports and policy documents were also charted, summarised, and integrated into the review findings.

### Stage 6: Expert consultation

Consultations took place with national experts from the Irish National Drug-Related Deaths Index (NDRDI) Steering Committee and the Technical Advisory Group of the National Office of Suicide Prevention, and international experts from the European Monitoring Centre for Drugs and Drug Addiction (EMCDDA), the World Health Organization (WHO), and key authors in the area of addiction research. These consultations, which were primarily by email, provided references for review and insights into international issues associated with factors for death by suicide in PWUD not previously found in the literature.

## Results

### Publication characteristics

Characteristics related to 152 publications were charted, with a final selection of 53 publications included in the scoping review.

The majority (n = 46, 87%) of publications included primary research, with an uncontrolled, retrospective study design [20, 21, 24, 42–84] (Table 2). The most common type of data sources used in primary research studies were drug treatment databases or national death indexes, followed by coroner’s records and medical records. Secondary research consisted of literature reviews or reports (n = 6, 11%) [23, 25, 85–88], and one editorial article [89].

Almost half (n = 22, 42%) of publications contained data from studies carried out in the European countries of Denmark, the United Kingdom, Spain, Norway, France, Slovenia, Scotland, Finland, Italy, Switzerland, and Sweden [20, 42–44, 48–51, 56, 59–63, 65, 66, 70, 82–84, 87, 89]. The next largest group were studies carried out in Australia (n = 11, 21%) [53–55, 57, 58, 71, 73, 74, 77, 78, 81], followed by the United States (US) (n = 7, 14%) [21, 24, 45, 47, 52, 67, 68]. Over three quarters (n = 40, 75%) of publications were published since 2013.

Thirty-six (68%) publications focused on PWUD as the population [20, 25, 54–84, 86–88], while the remaining 17 (32%) included outcomes for PWUD as a sub-group [21, 23, 24, 42–53, 85, 89]. Diagnostic inclusion criteria for drug use included a clinical diagnosis or clear history of drug use, corresponding International Classification of Diseases (ICD) codes, or inclusion in opioid agonist treatment registers. Interviews or survey methods, either of the person themselves or next of kin, were used in three (6%) publications to determine a participant’s drug use [47, 52, 54].

The definition of suicide varied across the publications, depending on the primary data source used (Table 2). The most common way to determine suicide was through use of ICD codes (n = 23, 43%), and coroner’s verdicts and autopsy findings (n = 14, 26%), followed by manner of death as recorded in general mortality registers (n = 7, 13%). Overall, there was limited in-depth analysis of explicit risk factors for suicide among PWUD, highlighting this as a gap in research.

### Drugs identified in publications exploring suicide in PWUD

The majority of publications included in the review focused on the use of a particular type of drug. Twenty-four publications examined opioid use, including the use of opioid agonist treatment (OAT) in PWUD. Two publications focused on cannabis use and mortality [55, 86], one on cocaine use disorder in patients with concurrent alcohol or opioid disorder [56], one on use of methamphetamines [57], one on opioid or amphetamine use [58], and one on new psychoactive substances (NPS) [59]. The remaining 24 publications included any type of DUD population [20, 21, 23, 29, 42–53, 60–64, 87, 89].

Overall, opioids were the most commonly reported drug type used by PWUD who died by suicide (n = 34, 64%), followed by cannabis (n = 10, 19%), and cocaine (n = 8, 15%). Polydrug use was linked to death by suicide in ten (19%) publications. A breakdown of the primary drugs linked to PWUD who died by suicide is provided in Table 3.

Where the suicide decedent used opioids, the concurrent presence of central nervous system depressants such as benzodiazepines, or antidepressants, was linked to increased risk of death by suicide in three publications [65, 66, 88]. The distinction between whether a person who used opioids was abusing prescription or illicit opioids was not made in most publications, although 15 studies focused on PWUD who were prescribed opioid agonist treatment such as methadone or buprenorphine [49, 67–78, 85]. A further two studies identified PWUD who misused licit or prescription opioids, including oxycodone and dihydrocodeine, and who died by suicide [66, 79].

The majority of publications presented aggregate data on suicide method in PWUD. Twelve studies provided specific data on the method of suicide [45, 48, 54, 55, 57–60, 62, 65, 74, 80]. Non-poisoning deaths, such as hangings or death by firearms, appeared more frequent than poisoning deaths in PWUD, in these particular publications.

**Table 2:** Characteristics of included publications

Type of evidence	Primary research		Review or report		Editorial	
No. of publications (Reference(s))	**46** [20, 21, 24, 41, 42, 44, 46–56, 58–64, 66–69, 71–88]		**6** [23, 25, 45, 57, 65, 70]		**1** [43]	
Origin of evidence	**Europe**	**Australia**	**United States**	**International**	**Asia**	**Canada**
	**22** [20, 41–44, 49–52, 58, 61–66, 68, 69, 74, 86–88]	**11** [54–56, 59, 60, 75, 77, 78, 81, 82, 85]	**7** [21, 24, 46, 48, 53, 71, 72]	**5** [23, 25, 45, 57, 70]	**5** [73, 76, 79, 80, 84]	**3** [47, 67, 83]
Primary data source	**Drug treatment database**	**National death index or suicide register**	**Medical records**	**Coronial or autopsy files**	**Other patient register**	**Veterans’ Health Administration database**
No. of publications (Reference(s))	**16** [20, 41, 55, 63, 67, 72–81, 85]	**13** [46–48, 50, 51, 53, 54, 56, 58–60, 82, 84]	**7** [21, 49, 64, 66, 86–88]	**5** [61, 62, 68, 69, 83]	**3** [42, 44, 52]	**2** [24, 71]
Main measurement of suicide risk	**Time to event data**	**Regression or multifactorial methods**	**CMR/SMR or PYLL**	**Descriptive statistics or cross-tabulation**	**Narrative synthesis**	**Meta-analysis**
No. of publications (Reference(s))	**16** [20, 24, 41, 47, 52, 55, 60, 63, 67, 71, 76, 78–82]	**12** [21, 44–46, 48, 50, 51, 53, 54, 56, 64, 84]	**12** [23, 58, 59, 66, 73–75, 77, 85–88]	**8** [42, 49, 61, 62, 68, 69, 72, 83]	**4** [25, 43, 65, 70]	**1** [57]

Abbreviations: CMR – crude mortality rate; SMR – standardised mortality ratio; PYLL – potential years of life lost

**Table 3:** Primary drug(s) identified in publications of PWUD who died by suicide

Drug	Opioids	Cannabis (Marijuana)	Cocaine	Sedatives/ Hypnotics	Amphetamines	Other illicit drugs	Polydrug use
No. of publications (Reference(s))	**34** [20, 23, 25, 26, 43–46, 49–54, 56–60, 62, 64, 65, 69, 70, 72–78, 81, 84, 85]	**10** [20, 24, 41, 46, 48, 52, 56, 57, 62, 64]	**8** [25, 51, 61, 63, 65, 69, 75, 79]	**7** [24, 50–52, 68–70]	**5** [24, 46, 59, 60, 62]	**3** [50, 61, 64]	**10** [20, 21, 23, 41, 46, 49, 51, 62, 64, 83]

**Table 4:** Factors associated with death by suicide in people who use drugs

Emergent theme	Sex	Mental health conditions	Periods of heightened vulnerability	Age profile	Stimulants, cannabis, or new psychoactive substances and method of death		Medical conditions
No. of publications (Reference(s))	**26** [21, 24, 25, 46–52, 54–56, 59, 60, 64–67, 76, 79–82, 85, 88]	**22** [20, 24, 25, 41–44, 47, 49, 50, 55, 59, 64, 68–71, 73, 78, 80, 83, 84]	**8** [43, 49, 63, 70, 74, 75, 80, 81]	**7** [20, 64–66, 68, 72, 74]	**7** [46, 48, 49, 56, 59, 61, 62]		**7** [21, 25, 44, 45, 49, 81, 83]
Emergent theme	**Dual diagnosis service provision**	**Homelessness**	**Incarceration**	**Intravenous (IV) drug use**		**Race/ethnicity**	
No. of publications (Reference(s))	**4** [43, 50, 56, 69]	**3** [43, 52, 54]	**3** [45, 49, 77]	**3** [23, 59, 67]		**2** [53, 86]	

### Factors associated with death by suicide in PWUD

Themes and associated publications are provided in Table 4.

#### Sex

Sex was reported as a candidate factor in 26 (53%) publications. Twelve were primary research studies [25, 45, 47, 53–55, 57, 58, 62, 64, 72, 77, 81], one was a technical report [87], and one was a narrative review [25].

Male sex was reported as a factor associated with death by suicide in 14 (54%) publications. Men who used drugs were more likely to die by suicide than women in 11 primary research studies [45, 53–55, 57, 58, 62, 64, 72, 73, 77]. Darke and Ross [25] reported a higher prevalence of death by suicide among men who used heroin relative to women who used heroin throughout the literature. The EMCDDA also reported that PWUD who died by suicide in Europe were predominantly male.

Cannabis use was identified as a risk factor for suicide in men in three publications [47, 55, 62]. Stimulants, such as amphetamines, were also more common among men who use drugs and died by suicide, than women [45, 57, 58, 62].

Thirteen (50%) of the 26 publications that reported on sex reported links with female sex and risk of death by suicide [21, 24, 46–51, 63, 75, 76, 78, 84]. While it is generally accepted men are at higher risk of death by suicide than women in the general population [3, 16], eight studies reported proportionally higher risk of suicide in women with DUD than in men with DUD [21, 24, 46, 47, 51, 75, 78, 84]. Onyeka et al. [63] identified a higher mean potential years of life lost (PYLL) due to suicide for women who used drugs than men who used drugs (44.9 years vs. 39.1), even though men had higher absolute numbers of deaths.

While Adams et al. [49] did not demonstrate an increased risk of death by suicide among women in comparison to men in their study, they identified higher proportions of sedative, hypnotic or anxiolytic-related disorders, and psychoactive substance use disorders among women who died by suicide than in men.

In the two studies that solely focused exclusively on women who used drugs, high rates of mental health problems were linked to risk of death by suicide [48, 76]. No studies focused exclusively on men. Of note is the lack of evidence in the area of trans and gender-diverse people who use drugs and die as a result of suicide.

#### Mental health conditions

Long-term SUD have been linked to mental health issues, and conversely, mental health conditions have been linked to increased levels of drug or alcohol use [90]. The term dual diagnosis, or the combined presence of a mental health problem and a substance use problem, may be used in practice as a diagnostic label [90]. There was a high prevalence of mental health problems among PWUD who died by suicide in the review. Twenty-two (42%) publications identified mental health as a candidate factor for death by suicide in this population [20, 24, 25, 42–44, 46, 48, 49, 54, 57, 62, 65–67, 69, 74, 76, 79, 80, 88, 89].

Depressive disorders were the most frequently cited comorbid mental health condition identified in the review. Thirteen publications identified high prevalence of depression or anxiety, a history of self-harm, or a previous suicide attempt among PWUD who died by suicide [25, 44, 48, 54, 57, 65, 66, 69, 74, 76, 79, 80, 88]. The prevalence of depressive disorders or history of suicide attempt in people who use opioids who later died by suicide was as high as 65% [69] and 89% [54] in two studies respectively, although both studies had low overall numbers of suicide.

One study identified that depression in people who exclusively used cannabis, resulted in a lower risk of dying by suicide in comparison to people with depression without any SUD [42].

Schizophrenia spectrum disorders (SSD) or a history of psychosis was reported in five publications [42, 46, 48, 57, 79]. For women with DUD, Zaheer et al. [46] reported that a subsequent diagnosis of any SSD was a risk factor for death by suicide in comparison to men with the same condition. Poor compliance with medications for SSD or psychosis was reported in two studies [48, 57]. It is unknown whether the psychosis reported in several studies was drug-induced, or related to SSD, but Darke et al. [57] hypothesised that use of methamphetamines may induce psychosis or exacerbate a pre-existing condition.

Other types of mental health conditions, including attention deficit hyperactivity disorder (ADHD), obsessive-compulsive disorder (OCD), bulimia nervosa, personality disorder, adjustment disorder, and post-traumatic stress disorder (PTSD) were also linked to risk of death by suicide in PWUD [43, 48, 49, 67, 79]. Interestingly, one study found no increased risk of all-cause mortality in dual diagnosis patients who were PWUD, but specific data were not provided for suicide and dual diagnosis risk [62].

#### Periods of heightened vulnerability

For PWUD, there were periods where risk of suicide was heightened. Eight (15%) publications addressed various vulnerable periods related to OAT administration and timing [70, 71, 76, 77, 88], healthcare attendance patterns [61, 89], or recent imprisonment [48].

Initiating and ceasing OAT was identified as a period where risk of death by suicide was increased [71, 88]. Poor retention of individuals in OAT was a risk factor for death by suicide in women [76]. Repeated unsuccessful episodes of OAT were also linked to increased risk of suicide [70, 77].

For PWUD who were attending mental health or addiction services, a loss of contact was observed in the period immediately before a person’s suicide [89], indicating that this was a candidate risk factor. In a record-linkage study of drug-related death and suicide after hospital discharge in PWUD in Scotland for the years 1996–2006, hospitalisation and discharge marked the start of a period of heightened vulnerability for PWUD with respect to non-poisoning suicide, with 51 of 269 non-poisoning suicides occurring while hospitalised or in the 28 day period after being discharged [61]. The authors suggest that in this cohort, hospital contact may represent a desperate call for help.

The initial months of being in prison were a vulnerable period for women who used drugs. In case studies of 13 women who died by suicide in prison in the UK between 1992 and 2001, ten (76.9%) of the 13 women died by suicide within two months of being imprisoned [48]. The women all had multiple problems or upheaval in the days and weeks prior to their deaths, including withdrawal from drugs, lack of contact with families, bereavement, and relationship problems outside and within prison. More than two thirds had also recently been relocated, often against their wishes and to other prison accommodation that they found less acceptable.

#### Age profile

Seven (11%) publications included an age profile as a candidate factor [20, 62, 63, 65, 68, 70, 87]. Death by suicide was linked to a younger age profile or higher number of PYLL in six publications [20, 62, 63, 65, 68, 87], although the definition of ‘younger age’ varied by study, from teens to PWUD aged in their forties. A technical report by the EMCCDA on drug-related mortality in Europe identified that PWUD who were in their teens and early 20s, were at greatest risk of suicide among PWUD across 14 European countries [87]. Stenbacka et al. [62] reported that nearly 20% of PWUD aged 24 years or younger in their longitudinal cohort study died by suicide.

Five studies focused on PWUD seeking treatment for opioid use [20, 63, 65, 68, 70]. One study involving people who used heroin, aged between 15 and 59 and seeking OAT treatment in Slovenia, found that older age at treatment entry was an important risk factor for death by suicide. The hazard risk for death by suicide was significantly higher in patients entering the cohort when older (HR = 1.08, 95% CI: 1.02–1.13, p = 0.003) [70].

#### Stimulants, cannabis, and new psychoactive substance (NPS) use and method of death

Where publications provided data on whether a suicide was a poisoning or non-poisoning, an emergent theme was the association of stimulants, cannabis, and NPS with violent deaths. Six (11%) publications identified these substances as possible candidate risk factors for violent, non-poisoning suicide deaths [45, 48, 55, 57, 59, 60].

The majority (85%) of suicide deaths in people who used methamphetamine in an Australian cohort study were violent suicide deaths. Zahra et al. [55] found that 92% of people who used cannabis and died by suicide in Australia, died by violent means. In a retrospective review of autopsy reports, Delaveris et al. [60] identified that the illicit drug toxicology profiles in non-poisoning suicides were more similar to homicide deaths than poisoning suicides. The toxicology profile of poisonings was more similar to accidental overdoses in PWUD. Cannabis was present in almost half of these suicides followed by amphetamines (35.3%), opioids (15.1%), and cocaine (8.4%). Mackenzie et al. [48] also identified a high prevalence of cocaine use in their case series of incarcerated women, all of whom died by violent method of suicide.

Elliott and Evans [59] identified that 17% of deaths where NPS were present were fatal hangings, with a further 5% other types of violent suicide. Cathinone drugs such as mephedrone, were more prevalent that other types of NPS in these suicides. However, it is important to note that this study did not compare NPS rates in non-poisoning suicides.

One study found a significant link between opioid use and suicide by firearms, but this was a study that solely focused on violent methods of suicide, and similarly to the study above, no comparative toxicology data for poisoning suicides were available [45].

#### Medical conditions

Blood borne viruses (BBV) were the most prevalent medical condition identified in the review. Four (8%) studies identified BBV status as a factor associated with suicide deaths in PWUD [25, 44, 77, 85]. Intravenous drug use increases the incidence of HIV and other BBV such as Hepatitis B and C, through high-risk practices such as sharing or reusing needles and syringes. BBV are known to precipitate chronic diseases and increase risk of premature mortality [77]. HIV infection in particular was linked to suicide and overdose among people who use heroin or other opioids [25, 77]. Vajdic et al. [77], in their study of 29,571 opioid-dependent people in Australia, found that risk of death by suicide increased with notification of HIV infection in bivariable analyses, but not multivariable analyses.

Two (4%) studies identified other medical conditions as candidate factors for death by suicide [21, 79]. Madadi et al. [79] found that a history of cancer and chronic pain were risk factors for death by suicide in a cohort of people who used opioids. The Charlson Comorbidity Index (CCI) was used by Lynch et al. [21] to measure non-psychiatric medical comorbidities, such as cancer and cardiovascular disease, in a case-control study of substance use disorders and suicide risk in the US general population. PWUD who died by suicide were more likely than controls to have a higher CCI score, indicating more severe medical illness at the time of death.

Mackenzie et al. [48] found that 38.5% of the women who died by suicide while incarcerated had a physical illness, such as epilepsy or asthma.

#### Dual diagnosis service provision

Issues in relation to dual diagnosis service provision and wider policies, were identified as contributory towards risk of death by suicide in four (8%) publications. The combined presence of a mental health problem and a substance use problem has previously been identified as a barrier to accessing treatment, where individuals are unable to access mental health services because of addiction, and vice versa [90].

Appleby [89] identified a high risk of suicide in PWUD who were dual diagnosis patients, and highlighted the fact that separation of services led to disrupted patterns of care and loss of contact with individuals prior to their suicide. Zahra et al. [55] identified the need for integration of mental health and addiction services in Australia, suggesting that it would be beneficial for professionals treating people with cannabis dependence to also screen individuals for suicide ideation, given their increased risk of suicide.

Untreated or inappropriately managed comorbid mental health issues in PWUD were suggestive of a lack of dual diagnosis service provision in two further cohort studies. In Denmark, men who were prescribed drugs used to treat addictive disorders (e.g. buprenorphine), who were previously diagnosed with reaction to severe stress and with adjustment disorder but who were not prescribed antidepressants, antipsychotics, or anxiolytics, had an 84% suicide risk using a machine learning model relative to the comparison group, consisting of a 5% random sample of individuals living in Denmark who had a diagnosis of SUD during the study period [49]. In a cohort of opioid users, use of hypnotics and sedatives were associated with increased risk of death by suicide in comparison to accidental overdose. Prescribed antidepressants were also more common in this group. This could suggest poorly medicated withdrawal symptoms in dual diagnosis patients [66], and highlights the complex medical and psychological needs of this group of people.

#### Homelessness

PWUD are overrepresented in homeless populations [91–93]. Homelessness was identified as a risk factor in three (6%) studies in the review, two of which were longitudinal cohort studies. Arnautovska et al. [53] compared all suicide deaths in homeless and non-homeless people over a 20-year period, and found that homelessness significantly increased the risk of death by suicide in PWUD, in comparison to PWUD in the non-homeless population (42.4% vs. 20.4%). There was a high degree of social isolation in this population. Feodor Nilsson et al. [51] found that drug use resulted in an elevated hazard ratio (HR) for suicide in both men and women who were homeless at any point during a period of ten years, with a higher risk estimate for women (HR = 3.1, 95% CI = 1.8–5.4) than men (HR = 2.2, 95% CI = 1.8–2.8).

In an editorial piece by Appleby [89], discussing suicide data collected by the UK Confidential Inquiry between 1996 and 1998, PWUD accounted for 49% of suicide deaths among homeless people, a higher proportion than reported in the two previous cohort studies.

#### Incarceration

Incarceration was identified as a candidate factor in three (6%) publications [48, 73, 85]. There was a clear link between the absence of OAT in prison, and increased risk of death by suicide [48, 85], with the provision of OAT strongly protective [73]. Modelling carried out by Degenhardt et al. [85] suggested that scaling the provision of OAT up to the levels advised by the WHO in prisons could potentially avert between 13.7% and 51.1% of suicide deaths in Kentucky, Kyiv, and Tehran (the three international locations used for analysis in their study).

#### Intravenous (IV) drug use

Three (6%) studies suggested that IV drug use, particularly poly IV drug use, was a candidate factor for death by suicide [23, 57, 64]. Heroin was the primary IV drug of use identified [23, 64], with methamphetamine [23, 57], cocaine [23, 64], and other opioids [23] also identified.

Darke et al. [57], found that 25% of people who used methamphetamine and died by suicide in an Australian cohort study, had a history of IV drug use, or were currently injecting drugs. Hayashi et al. [64] identified poly IV drug use in a cohort of people who were injecting drugs in Canada, with the age-adjusted rate ratio for suicide risk significantly increased in men, in comparison to women. Wilcox et al. [23], in their review of cohort studies, identified an SMR of 1373 (95% CI 1029–1796) for poly IV drug use, indicating that individuals with opioid use disorder and poly IV drug use bore an elevated risk for death by suicide. The authors noted that this risk was also higher than the risk of suicide associated with individuals suffering from alcoholism in their review.

#### Race/ethnicity

Race and ethnicity as a potential factor was not a common theme, but was identified in two cohort studies [52, 82]. In a study of people who used heroin and were accessing substance use treatment in Italy, those born outside of Italy (non-natives) were distinguished for their higher percentage of suicide deaths. The standardised mortality ratio (SMR) for suicide among people who used heroin and were non-natives was 13.25 (6.63–26.50) versus 4.88 (95% CI 3.82–6.24) for those born in Italy.

Willis et al. [52] found that cocaine use was four times more likely among African Americans than White Americans in their study of a nationally representative sample of death certificates. Cocaine use was associated with increased risk of death by suicide among African Americans, in comparison to White Americans who also used cocaine (OR 4.59, 95% CI 1.97–10.72).

Both of these studies used relatively dated data; data from Willis et al. [52] were drawn from a 1993 sample, while Pavarin et al. [82] used data from between 1975 and 2016.

### Protective factors against death by suicide in PWUD

Current attendance in OAT or other medication-assisted drug treatment was identified as a protective factor against death by suicide in eight (15%) publications [67, 68, 70, 73, 76, 77, 85, 88]. Methadone was the most common type of OAT identified, but buprenorphine-naloxone, and naltrexone were also identified as protective. However, as discussed above, the period immediately after initiating or ceasing OAT, was identified as a period of increased risk of suicide in PWUD [71, 88].

Exclusive cannabis use was also identified as a protective factor against suicide in people with comorbid depression [42].

## Discussion

The objectives of this scoping review were to explore the evidence on factors associated with death by suicide among PWUD, to identify gaps in knowledge for future research, and to inform suicide prevention policy and best practice guidelines for working with PWUD, where appropriate. The majority of the evidence reviewed on suicide among PWUD was primary research, originating in Europe, and were based on surveillance systems that captured epidemiological data and trends. Publications generally lacked in-depth analysis of explicit risk factors for death by suicide in PWUD. The most prevalent candidate factor explored was sex, with approximately half of the included publications presenting sex-segregated data for overall numbers of suicide in PWUD. Specific associated factors were commonly not segregated by sex although five studies provided information on the different types of drugs used by men and women who died by suicide [24, 47, 50, 51, 63].

Men account for the majority of completed suicides worldwide [3, 94]. This is thought to be related to male tendency for higher lethality methods of suicide, and the reluctance of men to seek help for depression or suicidal ideation [1, 94]. Freeman et al. [10] argue that suicide attempts in females may represent less of an intention to die, and more a desire to communicate distress or change their social environment. In this review, absolute numbers of death by suicide were higher for men with DUD than for women with DUD, where sex-segregated data were available. However, there was a higher proportion of suicide in women with DUD than men with DUD in several studies [21, 24, 46, 47, 51, 75, 78, 84].

The fact that only five studies reported sex-segregated data highlights a deficit in how studies describing suicide in PWUD report on sex and gender. Where this factor was reported, it was mainly related to biological sex rather than gender identity, resulting in a knowledge gap. A key element to improving the lives of women and girls worldwide is to address sex and gender inequalities, as highlighted in the United Nation’s Global Agenda for Sustainable Development [95]. It is essential to include sex and gender in public health research to bridge the gap in public health knowledge and to advance gender and sex equality.

Increased rates of death by suicide in female PWUD were linked to high rates of mental health problems, high prevalence of opioid and other central nervous depressant drug use, and poor retention in DUD treatment. Lynn et al. [96] identified opioids and antidepressants as the main drugs implicated in suicide drug poisonings among women, a similar finding to the drug profile in females identified in this review. This suggests that female PWUD with depressive disorders are a more vulnerable population than women with depression without a DUD. Although OAT was identified as a protective factor against death by suicide among people who use opioids, poor retention in OAT was associated with increased risk of death by suicide in women. Women with DUD may encounter barriers in accessing OAT in comparison to men, such as stigma, lack of social supports including childcare, and lack of knowledge of services [96–98]. Migrant women may be particularly isolated and unable to access treatment [97], with ethnicity also a candidate factor for suicide in this review.

Mental health conditions increased risk of death by suicide for both men and women who were PWUD, both through the conditions themselves, and through issues concerning dual diagnosis service provision. Previous research has suggested that drug use may exacerbate underlying risk of suicide, or interact with mental illness to increase risk of engaging in suicidal behaviours [8]. Darke and Ross [25] suggest that between a quarter and a third of people who use heroin meet the criteria for a life-time diagnosis of major depression, a figure much higher than levels seen in the general population. Depressive disorders were the most common type of mental health condition reported in publications in the review.

Historically, some mental health services did not accept PWUD for treatment, and some services dedicated to treating SUD may not be equipped to deal with dual diagnosis [90], compounding the mental health difficulties experienced by PWUD with dual diagnosis. Fragmented healthcare services may also make it difficult to recognise when PWUD decrease or change their service use pattern, a risk factor for death by suicide identified by some publications in the review. As PWUD also frequently experience social exclusion [87], also known to be a risk factor for suicide [99], healthcare services may represent an important point of contact. There is therefore a need for integrated addiction and mental health treatment to decrease the risk of suicide in this population [89, 90]. Integrated care models which were shown to improve health outcomes among people with dual diagnosis in clinical trials are complex and challenging to scale-up in real world settings [100]. However, integrated care pathways for people with a dual diagnosis is an important step to ensure provision of services for people with dual diagnosis involves a single care pathway. In Ireland, a new model of care for dual diagnosis, which was developed in partnership with key stakeholders, has recently been launched, which is welcoming [101].

Suicide by hanging has previously been reported as the most common method of suicide in 16 European countries [102]. Darke and Ross [25] identified that people who use heroin were unlikely to use poisoning by heroin as a method of suicide. Violent methods of suicide were more frequent than suicides by poisoning in this review, in publications that reported method of death.

Impulsivity and substance use has previously been associated with suicidal ideation and suicidal behaviour [103]. Stimulant drugs and cannabis were most frequently identified in non-poisoning suicides in the review. Delaveris et al. [60] reported that the non-poisoning suicide illicit drug toxicology profile among PWUD appeared similar to the homicide toxicology profile, whereas the toxicology profile of poisonings was more similar to accidental overdoses. Amphetamines, cannabis, and cocaine were more prevalent in non-poisoning suicide deaths and homicides, while opioids were the most prevalent drug in poisoning suicide deaths and accidental overdoses [60]. Aggression and psychosis prior to suicide was also seen in both men and women who used methamphetamine [57]. This suggests that risk-taking and violent behaviour, including violent methods of suicide, may be increased with the ingestion of stimulant drugs. This differs to the presentation for people who use opioids, with high rates of depression, and therefore may require a different approach to reduce risk of suicide in this population.

Two studies identified race as a risk factor for suicide among PWUD. In people of colour who use drugs, the roles of systemic racism and racial violence may contribute to disproportionate harm, including risk of suicide.

The factors associated with suicide in PWUD identified in this review are those that primarily occur at the level of the individual, although they may point to broader community or societal issues [104]. This was necessary, in order to address the aims of the review, however, we acknowledge that there is a large body of literature exploring the structural conditions in which suicide in PWUD may occur.

Peer-led user movements and advocacy work in the areas of decriminalisation of drugs and expansion of access to harm reduction and social care for PWUD relevant to the local context and needs [105], as well as transformative justice approaches which seek to address the impact of drug-related stigma and harm [106], may also be considered important approaches to suicide prevention [107].

### Considerations for policy and practice

PWUD are at increased risk of death by suicide in comparison to the general population. While this review has used a scoping review design to identify factors associated with death by suicide in this population, there is a need for more high-quality, prospective primary research studies on suicide that include explicit risk factors.

A large proportion of the existing evidence on suicide in PWUD is related to opioid use. People who use opioids are a group that are relatively easier to capture and study in comparison to people who use non-opioid drugs, due to OAT registers or other drug treatment databases. While opioids are still responsible for the majority of drug-related deaths, cannabis is the most used substance worldwide, cocaine production is at a record high, and seizures of amphetamine and methamphetamine have increased [101]. It is therefore important to consider non-opioid drugs and their impact on suicide risk, in light of changing drug production and consumption patterns globally. Further studies in this area are needed.

### Strengths and limitations

To the best of our knowledge, this is the first scoping review undertaken on factors associated with death by suicide in PWUD. A strength of this review was the inclusion of all publications, including peer-reviewed articles and grey literature, as well as consultation with experts in the area. However, a limitation to scoping reviews is the lack of in-depth quality appraisal of the evidence. The search was limited to publications in between 2000 and 2021; our results are up to date as of November 2021 only; therefore, it is possible that potentially relevant publications before 2000 or after November 2021 could have been missed. However, we included secondary research that cited older seminal literature, and so captured additional relevant publications in this manner.

We included only English language publications, which may not be representative of all the evidence. This was necessary in order to avoid introducing erroneous conclusions by including papers that were not thoroughly understood, particularly when the context of suicide was so important to the objectives of the review.

Much US literature, which overlapped with veteran research, was excluded due to the absence of explicit candidate factors for death by suicide in PWUD within the study samples. This resulted in an over-representation of publications from Europe and Australia in this review, and so findings may not be reflective of the sociocultural context of drug use in the US.

Similarly, people with HIV were over-represented in studies that explored IV drug use or BBV in the review. HIV infection may be a potential confounder for risk of death by suicide in PWUD, as this illness confers additional medical complications.

## Conclusions

The majority of data available on death by suicide among PWUD were extracted from epidemiological research, with limited in-depth analysis of explicit risk factors for suicide in this cohort. Opioids were the most prevalent drugs of use in PWUD who died by suicide, followed by cannabis and stimulant drugs. Violent methods of suicide were more prevalent in cannabis and stimulant users. Sex, age profile, comorbid medical conditions, mental health conditions, and inadequate dual diagnosis service provision, were factors associated with death by suicide in PWUD. To prevent suicide in PWUD, it is important to consider risk factors and type of drug use, and to tailor policies and practices accordingly.

### Electronic supplementary material

Below is the link to the electronic supplementary material.

Supplementary Material 1: Population, concept and outcome definitions

Supplementary Material 2: Search terms for databases

Supplementary Material 3: PRISMA-ScR checklist

Supplementary Material 4: Charting form for included publications

## Data Availability

All data generated or analysed during this study are included in this published article [and its supplementary information files].

## List of Abbreviations

ADHD: Attention deficit hyperactivity disorder
AIDS: Acquired Immune Deficiency Syndrome
BBV: Blood Borne Viruses
CCI: Charlson Comorbidity Index
CI: Confidence Interval
CINAHL: Cumulative Index to Nursing and Allied Health Literature
CMR: Crude Mortality Rate
DUD: Drug Use Disorder
EMCDDA: European Monitoring Centre for Drugs and Drug Addiction
HIV: Human Immunodeficiency Virus
HR: Hazard Ratio
ICD: International Classification of Diseases
IV: Intravenous
NDRDI: National Drug-Related Deaths Index
NPS: New Psychoactive Substances
OAT: Opioid Agonist Treatment
OCD: Obsessive-compulsive disorder
OR: Odds Ratio
PRISMA: Preferred Reporting Items for Systematic Reviews and Meta-Analyses
PRISMA-ScR: PRISMA Extension for Scoping Reviews
PTSD: Post-traumatic stress disorder
PWUD: People Who Use Drugs
PYLL: Potential Years of Life Lost
SMR: Standardised Mortality Rate
SSD: Schizophrenia Spectrum Disorder
SUD: Substance Use Disorder
UK: United Kingdom
