# Supplementary material for: Factors associated with suicide in people who use drugs: a scoping review
Source: BMC Psychiatry. 2023 Sep 5;23:655. doi: 10.1186/s12888-023-05131-x (PMC10478413; doi:10.1186/s12888-023-05131-x)
Supplement: Supplementary file 1 — Supplementary Material 1: Population, concept and outcome definitions [file 12888_2023_5131_MOESM1_ESM.docx]

**Appendix 2**

PCO definitions

**Population**

The review team note the variety of terms used to describe PWUD in this body of literature. For the purposes of the scoping review, PWUD is considered an umbrella phrase under which various terms indicative of problem drug use are subsumed, including, but not limited to, any of the following:

(a) People who use, misuse, or abuse drugs (including non-medical use of prescribed drugs and illicit drug use).

(b) People with a diagnosis of substance use disorder (SUD) / drug use disorder (DUD).

(c) People with drug dependence.

(d) People who are regular or ‘casual’ users of drugs.

(e) People who report recent drug use.

**Concept**

For the purposes of the scoping review, a candidate factor is defined as any characteristic, fixed or modifiable, that is associated with death by suicide. Given the wide range of possible candidate factors, the scope of the review was kept deliberately broad so that factor categories could emerge during the process of the systematic search and data charting. We anticipated that some evidence sources may not explicitly identify a correlate or predictor of suicide as a risk factor per se, and so decisions on the parameters of what constitutes a factor associated with suicide among PWUD were made iteratively as the team became increasingly familiar with the breadth and nature of the field.

**Outcome**

In relation to suicide as an outcome for PWUD, studies may refer to one or more of the following:

(a) Suicide, defined as “Different manners of non-natural death have different numbers of undetermined cases in terms of intent; for example, in a hanging or a shooting it is usually easy to differentiate between a suicide or a trauma (or a crime), while for drowning, traffic collisions or intoxication it is more difficult. Circumstantial findings, such as suicide notes, expressed intent or other findings such as self-inflicted cutting of the wrist followed by drowning, are suggestive of the intent” (E950-953, X60-84-ICD–International Classification of Disease–9 and 10).

(b) Undetermined suicide, defined as “When crime can be ruled out and it cannot be established whether the manner of death is a suicide or an accident, the manner of death is recorded as death of undetermined intent” (E980, Y10-34, ICD–International Classification of Disease–9 and 10).

(c) Probable suicide. All deaths with a diagnosis of suicide or undetermined suicide can be considered probable suicide deaths.
