# Supplementary material for: Factors associated with suicide in people who use drugs: a scoping review
Source: BMC Psychiatry. 2023 Sep 5;23:655. doi: 10.1186/s12888-023-05131-x (PMC10478413; doi:10.1186/s12888-023-05131-x)
Supplement: Supplementary file 2 — Supplementary Material 2: Search terms for databases [file 12888_2023_5131_MOESM2_ESM.docx]

**Appendix 3**

Search terms for databases

**Medline Final Search**

1. Suicide/

2. Suicide, Attempted/

3. Suicide, Completed/

4. "Cause of Death"/

5. suicide*.mp.

6. "completed suicide".mp.

7. "committed suicide".mp.

8. (risk adj3 suicide).mp.

9. "suicide risk".mp.

10. (death adj3 suicide).mp.

11. "probable suicide".mp.

12. "undetermined suicide".mp.

13. "suicid* intent*".mp.

14. (intent* adj4 (death or suicide or harm)).mp.

15. (intent* adj2 (overdose or poisoning)).mp.

16. (deliberate* adj2 (overdose or poisoning)).mp.

17. Substance-Related Disorders/

18. Amphetamine-Related Disorders/

19. Cocaine-Related Disorders/

20. Inhalant Abuse/

21. Marijuana Abuse/

22. Narcotic-Related Disorders/

23. Opioid-Related Disorders/

24. Heroin Dependence/

25. Morphine Dependence/

26. Opium Dependence/

27. Phencyclidine Abuse/

28. Substance Abuse, Intravenous/

29. Substance Abuse, Oral/

30. Drug Misuse/

31. Prescription Drug Misuse/

32. Prescription Drug Overuse/

33. Drug Users/

34. Illicit Drugs/

35. Controlled Substances/

36. (substance adj (use* or abuse* or misuse* or depend*)).mp.

37. (substance adj2 (disorder* or problem*)).mp.

38. (drug adj3 (use* or abuse* or misuse* or depend* or addict*)).mp.

39. "drug use disorder*".mp.

40. "recent drug use".mp.

41. "prescription drug*".mp.

42. (prescription drug adj (use* or abuse* or misuse* or depend* or addict*)).mp.

43. "nonprescription drug*".mp.

44. (nonprescription drug adj (use* or abuse* or misuse* or depend* or addict*)).mp.

45. "non-prescription drug*".mp.

46. (non-prescription drug adj3 (use* or abuse* or misuse* or depend* or addict*)).mp.

47. "illicit drug*".mp.

48. (illicit drug adj (use* or abuse* or misuse* or depend* or addict*)).mp.

49. (injection drug adj (use* or abuse* or misuse* or depend* or addict*)).mp.

50. (injecting drug adj (use* or abuse* or misuse* or depend* or addict*)).mp.

51. "intravenous drug*".mp.

52. "intravenous drug use".mp.

53. (people adj3 drugs).mp.

54. (substance adj3 patients).mp.

55. "addictive substance*".mp.

56. (polydrug adj (use* or abuse* or depend* or addict*)).mp.

57. (polysubstance adj (use* or abuse* or depend* or addict*)).mp.

58. (cannabis adj2 (disorder or use* or abuse* or misuse* or depend* or addict*)).mp.

59. (marijuana adj2 (disorder or use* or abuse* or misuse* or depend* or addict*)).mp.

60. (narcotic adj (use* or abuse* or misuse* or depend* or addict*)).mp.

61. (opiate adj2 (disorder or use* or abuse* or misuse* or depend* or addict*)).mp.

62. (opioid adj2 (disorder or use* or abuse* or misuse* or depend* or addict*)).mp.

63. "opioid dependent people".mp.

64. (heroin adj2 (disorder or use* or abuse* or misuse* or depend* or addict*)).mp.

65. (cocaine adj2 (disorder or use* or abuse* or misuse* or depend* or addict*)).mp.

66. (solvent adj (use* or abuse*)).mp.

67. (inhalant adj (use* or abuse*)).mp.

68. (benzodiazepine adj (use* or abuse* or misuse* or depend* or addict*)).mp.

69. (sedative adj (use* or abuse* or misuse* or depend* or addict*)).mp.

70. (barbiturate adj (use* or abuse* or misuse* or depend* or addict*)).mp.

71. (steroid adj (use* or abuse* or misuse* or depend* or addict*)).mp.

72. "non-medical use of drugs".mp.

73. (amphetamine* adj (use* or abuse* or misuse* or depend* or addict*)).mp.

74. (methamphetamine* adj (use* or abuse* or misuse* or depend* or addict*)).mp.

75. 1 or 2 or 3 or 4 or 5 or 6 or 7 or 8 or 9 or 10 or 11 or 12 or 13 or 14 or 15 or 16

76. 17 or 18 or 19 or 20 or 21 or 22 or 23 or 24 or 25 or 26 or 27 or 28 or 29 or 30 or 31 or 32 or 33 or 34 or 35 or 36 or 37 or 38 or 39 or 40 or 41 or 42 or 43 or 44 or 45 or 46 or 47 or 48 or 49 or 50 or 51 or 52 or 53 or 54 or 55 or 56 or 57 or 58 or 59 or 60 or 61 or 62 or 63 or 64 or 65 or 66 or 67 or 68 or 69 or 70 or 71 or 72 or 73 or 74

77. 75 and 76

78. exp animals/ not humans.sh.

79. 77 not 78

80. limit 79 to yr="2000 -Current"

**CINAHL Final Search**

**S1** (MH "Suicide") OR (MH "Suicide, Attempted") OR (MH "Self-Injurious Behavior") OR (MH "Injuries, Self-Inflicted") OR (MH "Mortality") OR (MH "Cause of Death")

[OR]

**S2** ( suicide* OR "completed suicide" OR "committed suicide" OR (risk N3 suicide) OR "suicide risk" OR (death N3 suicide) OR "probable suicide" OR "undetermined suicide" OR "suicid* intent*" OR (intent* N4 (death or suicide or harm)) OR (intent* N2 (overdose or poisoning)) OR (deliberate* N2 (overdose or poisoning) )

[AND]

**S3** ( (MH "Substance Use Disorders") OR (MH "Substance Abuse") OR (MH "Inhalant Abuse") OR (MH "Substance Abuse, Intravenous") OR (MH "Substance Abuse, Perinatal") OR (MH "Substance Dependence") OR (MH "Substance Abusers") OR (MH "Intravenous Drug Users") OR (MH "Amphetamines") OR (MH "Amphetamine") OR (MH "Methamphetamine") OR (MH "Cannabis") OR (MH "Cannabinoids") OR (MH "Analgesics") OR (MH "Analgesics, Opioid") OR (MH "Heroin") OR (MH "Alkaloids") OR (MH "Cocaine") OR (MH "Crack Cocaine") OR (MH "Narcotics") OR (MH "Narcotic Antagonists") OR (MH "Anesthetics") OR (MH "Drugs") OR (MH "Controlled Substances") OR (MH "Counterfeit Drugs") OR (MH "Designer Drugs") OR (MH "Drugs, Non-Prescription") OR (MH "Drugs, Off-Label") OR (MH "Drugs, Prescription") OR (MH "Street Drugs") OR (MH "Substandard Drugs") OR (MH "Synthetic Drugs") OR (MH "Hypnotics and Sedatives") OR (MH "Psychotropic Drugs") OR (MH "Hallucinogens") OR (MH "Tranquilizing Agents") OR (MH "Antianxiety Agents, Benzodiazepine") OR (MH "Muscle Relaxants, Central") OR (MH "Barbiturates") OR (MH "Solvents") OR (MH "Dopamine Agents") OR (MH "Adrenergic Agents") OR (MH "Serotonin Agents") OR (MH "Steroids") )

[OR]

**S4** ( (substance N0 (use* or abuse* or misuse* or depend*)) OR (substance N2 (disorder* or problem*)) OR (drug N3 (use* or abuse* or misuse* or depend* or addict*)) OR "drug use disorder*" OR "recent drug use" OR "prescription drug*" OR (prescription drug N0 (use* or abuse* or misuse* or depend* or addict*)) OR "nonprescription drug*" OR (nonprescription drug N0 (use* or abuse* or misuse* or depend* or addict*)) OR "non-prescription drug*" OR (non-prescription drug N3 (use* or abuse* or misuse* or depend* or addict*)) OR "illicit drug*" OR (illicit drug N0 (use* or abuse* or misuse* or depend* or addict*)) OR (injection drug N0 (use* or abuse* or misuse* or depend* or addict*)) OR (injecting drug N0 (use* or abuse* or misuse* or depend* or addict*)) OR "intravenous drug*" OR "intravenous drug use" OR (people N3 drugs) OR (substance N3 patients) OR "addictive substance*" OR "psychotropic drug*" OR "psychoactive drug*" OR (polydrug N0 (use* or abuse* or depend* or addict*)) OR (polysubstance N0 (use* or abuse* or depend* or addict*)) OR "cannabinoids" OR "cannabis" OR (cannabis N2 (disorder or use* or abuse* or misuse* or depend* or addict*)) OR "marijuana" OR (marijuana N2 (disorder or use* or abuse* or misuse* or depend* or addict*)) OR "narcotics" OR (narcotic N0 (use* or abuse* or misuse* or depend* or addict*)) OR "opiate*" OR (opiate N2 (disorder or use* or abuse* or misuse* or depend* or addict*)) OR "opioid*" OR (opioid N2 (disorder or use* or abuse* or misuse* or depend* or addict*)) OR “opioid dependent people” OR "heroin" OR (heroin N2 (disorder or use* or abuse* or misuse* or depend* or addict*)) OR "cocaine" OR "crack cocaine" OR (cocaine N2 (disorder or use* or abuse* or misuse* or depend* or addict*)) OR "solvent*" OR (solvent N0 (use* or abuse*)) OR "inhalant*" OR (inhalant N0 (use* or abuse*)) OR "benzodiazepine*" OR (benzodiazepine N0 (use* or abuse* or misuse* or depend* or addict*)) OR "hypnotics" OR "hallucinogens" OR "sedative*" OR (sedative N0 (use* or abuse* or misuse* or depend* or addict*)) OR "barbiturate*" OR (barbiturate N0 (use* or abuse* or misuse* or depend* or addict*)) OR "steroids" OR (steroid N0 (use* or abuse* or misuse* or depend* or addict*)) OR "non-medical use of drugs" OR (amphetamine* N0 (use* or abuse* or misuse* or depend* or addict*)) OR (methamphetamine* N0 (use* or abuse* or misuse* or depend* or addict*)) )

**S5** S1 OR S2

**S6** S3 OR S4

**S7** S5 AND S6

**S8** MH animals+

**S9** MH (animal studies)

**S10** TI (animal model*)

**S11** S8 OR S9 OR S10

**S12** MH (human)

**S13** S11 NOT S12

**S14** S7 NOT S13

**Limiters** - Published Date: 20000101-20201231

**Expanders** - Apply equivalent subjects

**Search** **modes** - Boolean/Phrase

**PsychINFO Final Search**

1. Suicide/

2. Attempted Suicide/

3. Suicidality/

4. Psychological Autopsy/

5. Suicidology/

6. Mortality Rate/

7. Mortality Risk/

8. suicide*.mp.

9. "completed suicide".mp.

10. "committed suicide".mp.

11. (risk adj3 suicide).mp.

12. "suicide risk".mp.

13. (death adj3 suicide).mp.

14. "probable suicide".mp.

15. "undetermined suicide".mp.

16. "suicid* intent*".mp.

17. (intent* adj4 (death or suicide or harm)).mp.

18. (intent* adj2 (overdose or poisoning)).mp.

19. (deliberate* adj2 (overdose or poisoning)).mp.

20. "Substance Use Disorder"/

21. Drug Addiction/

22. "Cannabis Use Disorder"/

23. Drug Abuse/

24. Inhalant Abuse/

25. Polydrug Abuse/

26. Drug Dependency/

27. "Opioid Use Disorder"/

28. Heroin Addiction/

29. Morphine Dependence/

30. Drug Usage/

31. Intravenous Drug Usage/

32. Prescription Drug Misuse/

33. (substance adj (use* or abuse* or misuse* or depend*)).mp.

34. (substance adj2 (disorder* or problem*)).mp.

35. (drug adj3 (use* or abuse* or misuse* or depend* or addict*)).mp.

36. "drug use disorder*".mp.

37. "recent drug use".mp.

38. "prescription drug*".mp.

39. (prescription drug adj (use* or abuse* or misuse* or depend* or addict*)).mp.

40. "nonprescription drug*".mp.

41. (nonprescription drug adj (use* or abuse* or misuse* or depend* or addict*)).mp.

42. "non-prescription drug*".mp.

43. (non-prescription drug adj3 (use* or abuse* or misuse* or depend* or addict*)).mp.

44. "illicit drug*".mp.

45. (illicit drug adj (use* or abuse* or misuse* or depend* or addict*)).mp.

46. (injection drug adj (use* or abuse* or misuse* or depend* or addict*)).mp.

47. (injecting drug adj (use* or abuse* or misuse* or depend* or addict*)).mp.

48. "intravenous drug*".mp.

49. "intravenous drug use".mp.

50. (people adj3 drugs).mp.

51. (substance adj3 patients).mp.

52. "addictive substance*".mp.

53. (polydrug adj (use* or abuse* or depend* or addict*)).mp.

54. (polysubstance adj (use* or abuse* or depend* or addict*)).mp.

55. (cannabis adj2 (disorder or use* or abuse* or misuse* or depend* or addict*)).mp.

56. (marijuana adj2 (disorder or use* or abuse* or misuse* or depend* or addict*)).mp.

57. (narcotic adj (use* or abuse* or misuse* or depend* or addict*)).mp.

58. (opiate adj2 (disorder or use* or abuse* or misuse* or depend* or addict*)).mp.

59. (opioid adj2 (disorder or use* or abuse* or misuse* or depend* or addict*)).mp.

60. "opioid dependent people".mp.

61. (heroin adj2 (disorder or use* or abuse* or misuse* or depend* or addict*)).mp.

62. (cocaine adj2 (disorder or use* or abuse* or misuse* or depend* or addict*)).mp.

63. (solvent adj (use* or abuse*)).mp.

64. (inhalant adj (use* or abuse*)).mp.

65. (benzodiazepine adj (use* or abuse* or misuse* or depend* or addict*)).mp.

66. (sedative adj (use* or abuse* or misuse* or depend* or addict*)).mp.

67. (barbiturate adj (use* or abuse* or misuse* or depend* or addict*)).mp.

68. (steroid adj (use* or abuse* or misuse* or depend* or addict*)).mp.

69. "non-medical use of drugs".mp.

70. (amphetamine* adj (use* or abuse* or misuse* or depend* or addict*)).mp.

71. (methamphetamine* adj (use* or abuse* or misuse* or depend* or addict*)).mp.

72. 1 or 2 or 3 or 4 or 5 or 6 or 7 or 8 or 9 or 10 or 11 or 12 or 13 or 14 or 15 or 16 or 17 or 18 or 19

73. 20 or 21 or 22 or 23 or 24 or 25 or 26 or 27 or 28 or 29 or 30 or 31 or 32 or 33 or 34 or 35 or 36 or 37 or 38 or 39 or 40 or 41 or 42 or 43 or 44 or 45 or 46 or 47 or 48 or 49 or 50 or 51 or 52 or 53 or 54 or 55 or 56 or 57 or 58 or 59 or 60 or 61 or 62 or 63 or 64 or 65 or 66 or 67 or 68 or 69 or 70 or 71

74. 72 and 73

75. exp animals/ not humans.sh.

76. 74 not 75

77. limit 76 to yr="2000 -Current"

**SOCIndex Final Search**

**S1** ( (MH "SUICIDE") OR (MH "SUICIDE risk factors") OR (MH "ATTEMPTED suicide") OR (MH "SUICIDE, Attempted") OR (MH "SUICIDE attempts") OR (MH "SELF-injurious behavior") OR (MH "SELF-mutilation") OR (MH "MORTALITY") )

**S2 TI** ( (suicide* OR "completed suicide" OR "committed suicide" OR (risk N3 suicide) OR "suicide risk" OR (death N3 suicide) OR "probable suicide" OR "undetermined suicide" OR "suicid* intent*" OR (dying N3 "drug overdose") OR (intent* N4 (death or suicide or harm)) OR (intent* N2 (overdose or poisoning)) OR (deliberate* N2 (overdose or poisoning)) ) OR **AB** ( (suicide* OR "completed suicide" OR "committed suicide" OR (risk N3 suicide) OR "suicide risk" OR (death N3 suicide) OR "probable suicide" OR "undetermined suicide" OR "suicid* intent*" OR (dying N3 "drug overdose") OR (intent* N4 (death or suicide or harm)) OR (intent* N2 (overdose or poisoning)) OR (deliberate* N2 (overdose or poisoning)) ) OR **KW** ( (suicide* OR "completed suicide" OR "committed suicide" OR (risk N3 suicide) OR "suicide risk" OR (death N3 suicide) OR "probable suicide" OR "undetermined suicide" OR "suicid* intent*" OR (dying N3 "drug overdose") OR (intent* N4 (death or suicide or harm)) OR (intent* N2 (overdose or poisoning)) OR (deliberate* N2 (overdose or poisoning)) )

**S3** ( (MH "SUBSTANCE-induced disorders") OR (MH "SUBSTANCE abuse") OR (MH "CHURCH & substance abuse") OR (MH "DRUG abuse") OR (MH "AMPHETAMINE abuse") OR (MH "COCAINE abuse") OR (MH "DRUG addiction") OR (MH "DRUGS & sex") OR (MH "HEROIN abuse") OR (MH "INTRAVENOUS drug abuse") OR (MH "MARIJUANA abuse") OR (MH "MEDICATION abuse") OR (MH "KETAMINE abuse") OR (MH "OXYCODONE abuse") OR (MH "METHADONE abuse") OR (MH "SEDATIVE abuse") OR (MH "INHALANT abuse") OR (MH "AEROSOL sniffing") OR (MH "GLUE sniffing") OR (MH "PAINT sniffing") OR (MH "SUBSTANCE abuse diagnosis") OR (MH "SUBSTANCE abuse relapse") OR (MH "SUBSTANCE abuse -- Psychological aspects") OR (MH "SUBSTANCE abuse -- Research") OR (MH "SUBSTANCE abuse -- Risk factors") OR (MH "DRUG abuse risk factors") OR (MH "SUBSTANCE abuse -- social aspects") OR (MH "SUBSTANCE abuse treatment") OR (MH "DRUG abuse treatment") OR (MH "TREATMENT of drug addiction") OR (MH "BLACK people -- Substance use") OR (MH "BLACK people & drugs") OR (MH "CHILDREN -- Substance use") OR (MH "CHILDREN & drugs") OR (MH "EMPLOYEES -- Substance use") OR (MH "MEN -- Substance use") OR (MH "PRISONERS -- Substance use") OR (MH "PRISONERS -- Drug use") OR (MH "VETERANS -- Substance use") OR (MH "WOMEN -- Substance use") OR (MH "PREGNANT women -- Substance use") OR (MH "WOMEN & drugs") OR (MH "YOUTH -- Substance use") OR (MH "YOUTH & drugs") OR (MH "YOUNG adults & drugs") OR (MH "YOUNG adults -- Substance use") OR (MH "NATIVE Americans -- Substance use") OR (MH "NATIVE Americans -- Drug use") OR (MH "TEENAGERS -- Substance use") OR (MH "STUDENTS -- Substance use") OR (MH "HIGH school students -- Substance use") OR (MH "COLLEGE students -- Substance use") OR (MH "DRUG abusers") OR (MH "DRUG addicts") OR (MH "PSYCHOLOGY of drug abusers") OR (MH "PSYCHOLOGY of drug addiction") OR (MH "PSYCHOLOGY of drug addicts") OR (MH "DRUG addiction risk factors") OR (MH "AGE factors in substance abuse") OR (MH "DRUGS") OR (MH "CONTROLLED drugs") OR (MH "DRUGS of abuse") OR (MH "CLUB drugs") OR (MH "ECSTACY (Drug)") OR (MH "DESIGNER drugs") OR (MH "MARIJUANA") OR (MH "NARCOTICS") OR (MH "COCAINE") OR (MH "CRACK cocaine") OR (MH "HASHISH") OR (MH "OXYCODINE") OR (MH " NONPRESCRIPTION drugs") OR (MH "COCAINE abuse") OR (MH "COCAINE & psychology") OR (MH "MARIJUANA abuse") OR (MH "MARIJUANA -- Social aspects") OR (MH "HALLUCINOGENIC drugs") OR (MH "LSD (Drug)") OR (MH "HEROIN addicts") OR (MH "MEDICATION abusers") OR (MH "OFF-label use (Drugs)") OR (MH "PSYCHIATRIC drugs") )

**S4 TI** ( (substance N0 (use* or abuse* or misuse* or depend*)) OR (substance N2 (disorder* or problem*)) OR (drug N3 (use* or abuse* or misuse* or depend* or addict*)) OR "drug use disorder*" OR "recent drug use" OR "prescription drug*" OR (prescription drug N0 (use* or abuse* or misuse* or depend* or addict*)) OR "nonprescription drug*" OR (nonprescription drug N0 (use* or abuse* or misuse* or depend* or addict*)) OR "non-prescription drug*" OR (non-prescription drug N3 (use* or abuse* or misuse* or depend* or addict*)) OR "illicit drug*" OR (illicit drug N0 (use* or abuse* or misuse* or depend* or addict*)) OR (injection drug N0 (use* or abuse* or misuse* or depend* or addict*)) OR (injecting drug N0 (use* or abuse* or misuse* or depend* or addict*)) OR "intravenous drug*" OR "intravenous drug use" OR (people N3 drugs) OR (substance N3 patients) OR "addictive substance*" OR "psychotropic drug*" OR "psychoactive drug*" OR (polydrug N0 (use* or abuse* or depend* or addict*)) OR (polysubstance N0 (use* or abuse* or depend* or addict*)) OR "cannabinoids" OR "cannabis" OR (cannabis N2 (disorder or use* or abuse* or misuse* or depend* or addict*)) OR "marijuana" OR (marijuana N2 (disorder or use* or abuse* or misuse* or depend* or addict*)) OR "narcotics" OR (narcotic N0 (use* or abuse* or misuse* or depend* or addict*)) OR "opiate*" OR (opiate N2 (disorder or use* or abuse* or misuse* or depend* or addict*)) OR "opioid*" OR (opioid N2 (disorder or use* or abuse* or misuse* or depend* or addict*)) OR “opioid dependent people” OR "heroin" OR (heroin N2 (disorder or use* or abuse* or misuse* or depend* or addict*)) OR "cocaine" OR "crack cocaine" OR (cocaine N2 (disorder or use* or abuse* or misuse* or depend* or addict*)) OR "solvent*" OR (solvent N0 (use* or abuse*)) OR "inhalant*" OR (inhalant N0 (use* or abuse*)) OR "benzodiazepine*" OR (benzodiazepine N0 (use* or abuse* or misuse* or depend* or addict*)) OR "hypnotics" OR "hallucinogens" OR "sedative*" OR (sedative N0 (use* or abuse* or misuse* or depend* or addict*)) OR "barbiturate*" OR (barbiturate N0 (use* or abuse* or misuse* or depend* or addict*)) OR "steroids" OR (steroid N0 (use* or abuse* or misuse* or depend* or addict*)) OR "non-medical use of drugs" OR (amphetamine* N0 (use* or abuse* or misuse* or depend* or addict*)) OR (methamphetamine* N0 (use* or abuse* or misuse* or depend* or addict*)) ) OR **AB** ( (substance N0 (use* or abuse* or misuse* or depend*)) OR (substance N2 (disorder* or problem*)) OR (drug N3 (use* or abuse* or misuse* or depend* or addict*)) OR "drug use disorder*" OR "recent drug use" OR "prescription drug*" OR (prescription drug N0 (use* or abuse* or misuse* or depend* or addict*)) OR "nonprescription drug*" OR (nonprescription drug N0 (use* or abuse* or misuse* or depend* or addict*)) OR "non-prescription drug*" OR (non-prescription drug N3 (use* or abuse* or misuse* or depend* or addict*)) OR "illicit drug*" OR (illicit drug N0 (use* or abuse* or misuse* or depend* or addict*)) OR (injection drug N0 (use* or abuse* or misuse* or depend* or addict*)) OR (injecting drug N0 (use* or abuse* or misuse* or depend* or addict*)) OR "intravenous drug*" OR "intravenous drug use" OR (people N3 drugs) OR (substance N3 patients) OR "addictive substance*" OR "psychotropic drug*" OR "psychoactive drug*" OR (polydrug N0 (use* or abuse* or depend* or addict*)) OR (polysubstance N0 (use* or abuse* or depend* or addict*)) OR "cannabinoids" OR "cannabis" OR (cannabis N2 (disorder or use* or abuse* or misuse* or depend* or addict*)) OR "marijuana" OR (marijuana N2 (disorder or use* or abuse* or misuse* or depend* or addict*)) OR "narcotics" OR (narcotic N0 (use* or abuse* or misuse* or depend* or addict*)) OR "opiate*" OR (opiate N2 (disorder or use* or abuse* or misuse* or depend* or addict*)) OR "opioid*" OR (opioid N2 (disorder or use* or abuse* or misuse* or depend* or addict*)) OR “opioid dependent people” OR "heroin" OR (heroin N2 (disorder or use* or abuse* or misuse* or depend* or addict*)) OR "cocaine" OR "crack cocaine" OR (cocaine N2 (disorder or use* or abuse* or misuse* or depend* or addict*)) OR "solvent*" OR (solvent N0 (use* or abuse*)) OR "inhalant*" OR (inhalant N0 (use* or abuse*)) OR "benzodiazepine*" OR (benzodiazepine N0 (use* or abuse* or misuse* or depend* or addict*)) OR "hypnotics" OR "hallucinogens" OR "sedative*" OR (sedative N0 (use* or abuse* or misuse* or depend* or addict*)) OR "barbiturate*" OR (barbiturate N0 (use* or abuse* or misuse* or depend* or addict*)) OR "steroids" OR (steroid N0 (use* or abuse* or misuse* or depend* or addict*)) OR "non-medical use of drugs" OR (amphetamine* N0 (use* or abuse* or misuse* or depend* or addict*)) OR (methamphetamine* N0 (use* or abuse* or misuse* or depend* or addict*)) ) OR **KW** ( (substance N0 (use* or abuse* or misuse* or depend*)) OR (substance N2 (disorder* or problem*)) OR (drug N3 (use* or abuse* or misuse* or depend* or addict*)) OR "drug use disorder*" OR "recent drug use" OR "prescription drug*" OR (prescription drug N0 (use* or abuse* or misuse* or depend* or addict*)) OR "nonprescription drug*" OR (nonprescription drug N0 (use* or abuse* or misuse* or depend* or addict*)) OR "non-prescription drug*" OR (non-prescription drug N3 (use* or abuse* or misuse* or depend* or addict*)) OR "illicit drug*" OR (illicit drug N0 (use* or abuse* or misuse* or depend* or addict*)) OR (injection drug N0 (use* or abuse* or misuse* or depend* or addict*)) OR (injecting drug N0 (use* or abuse* or misuse* or depend* or addict*)) OR "intravenous drug*" OR "intravenous drug use" OR (people N3 drugs) OR (substance N3 patients) OR "addictive substance*" OR "psychotropic drug*" OR "psychoactive drug*" OR (polydrug N0 (use* or abuse* or depend* or addict*)) OR (polysubstance N0 (use* or abuse* or depend* or addict*)) OR "cannabinoids" OR "cannabis" OR (cannabis N2 (disorder or use* or abuse* or misuse* or depend* or addict*)) OR "marijuana" OR (marijuana N2 (disorder or use* or abuse* or misuse* or depend* or addict*)) OR "narcotics" OR (narcotic N0 (use* or abuse* or misuse* or depend* or addict*)) OR "opiate*" OR (opiate N2 (disorder or use* or abuse* or misuse* or depend* or addict*)) OR "opioid*" OR (opioid N2 (disorder or use* or abuse* or misuse* or depend* or addict*)) OR “opioid dependent people” OR "heroin" OR (heroin N2 (disorder or use* or abuse* or misuse* or depend* or addict*)) OR "cocaine" OR "crack cocaine" OR (cocaine N2 (disorder or use* or abuse* or misuse* or depend* or addict*)) OR "solvent*" OR (solvent N0 (use* or abuse*)) OR "inhalant*" OR (inhalant N0 (use* or abuse*)) OR "benzodiazepine*" OR (benzodiazepine N0 (use* or abuse* or misuse* or depend* or addict*)) OR "hypnotics" OR "hallucinogens" OR "sedative*" OR (sedative N0 (use* or abuse* or misuse* or depend* or addict*)) OR "barbiturate*" OR (barbiturate N0 (use* or abuse* or misuse* or depend* or addict*)) OR "steroids" OR (steroid N0 (use* or abuse* or misuse* or depend* or addict*)) OR "non-medical use of drugs" OR (amphetamine* N0 (use* or abuse* or misuse* or depend* or addict*)) OR (methamphetamine* N0 (use* or abuse* or misuse* or depend* or addict*)) )

**S5** S1 OR S2

**S6** S3 OR S4

**S7** S5 AND S6

**S8** S5 AND S6

**Limiters** - Date of Publication: **20000101-20201231**
Expanders - Apply equivalent subjects
Search modes - Boolean/Phrase

**Cochrane Database of Systematic Reviews Final Search**

#1 MeSH descriptor: [Suicide] this term only

#2 MeSH descriptor: [Suicide, Completed] this term only

#3 MeSH descriptor: [Suicide, Attempted] this term only

#4 MeSH descriptor: [Mortality] this term only

#5 MeSH descriptor: [Cause of Death] this term only

#6 MeSH descriptor: [Self-Injurious Behavior] this term only

#7 MeSH descriptor: [Self Mutilation] this term only

#8 MeSH descriptor: [Fatal Outcome] this term only

#9 MeSH descriptor: [Mortality, Premature] this term only

#10 MeSH descriptor: [Drug Overdose] this term only

#11 (suicide*) (Word variations have been searched)

#12 ("completed suicide") (Word variations have been searched)

#13 ("committed suicide") (Word variations have been searched)

#14 (risk adj3 suicide) (Word variations have been searched)

#15 ("suicide risk") (Word variations have been searched)

#16 (death adj3 suicide) (Word variations have been searched)

#17 ("probable suicide") (Word variations have been searched)

#18 ("undetermined suicide") (Word variations have been searched)

#19 ("suicid* intent*") (Word variations have been searched)

#20 (intent* adj4 (death or suicide or harm)) (Word variations have been searched)

#21 (intent* adj2 (overdose or poisoning)) (Word variations have been searched)

#22 (deliberate* adj2 (overdose or poisoning)) (Word variations have been searched)

#23 MeSH descriptor: [Substance-Related Disorders] this term only

#24 MeSH descriptor: [Amphetamine-Related Disorders] this term only

#25 MeSH descriptor: [Cocaine-Related Disorders] this term only

#26 MeSH descriptor: [Inhalant Abuse] this term only

#27 MeSH descriptor: [Marijuana Abuse] this term only

#28 MeSH descriptor: [Narcotic-Related Disorders] this term only

#29 MeSH descriptor: [Opioid-Related Disorders] this term only

#30 MeSH descriptor: [Heroin Dependence] this term only

#31 MeSH descriptor: [Morphine Dependence] this term only

#32 MeSH descriptor: [Opium Dependence] this term only

#33 MeSH descriptor: [Phencyclidine Abuse] this term only

#34 MeSH descriptor: [Substance Abuse, Intravenous] this term only

#35 MeSH descriptor: [Substance Abuse, Oral] this term only

#36 MeSH descriptor: [Drug Misuse] this term only

#37 MeSH descriptor: [Prescription Drug Misuse] this term only

#38 MeSH descriptor: [Prescription Drug Overuse] this term only

#39 MeSH descriptor: [Prescription Drugs] this term only

#40 MeSH descriptor: [Drug Users] this term only

#41 MeSH descriptor: [Illicit Drugs] this term only

#42 MeSH descriptor: [Crack Cocaine] this term only

#43 MeSH descriptor: [Analgesics] this term only

#44 MeSH descriptor: [Narcotics] this term only

#45 MeSH descriptor: [Analgesics, Opioid] this term only

#46 MeSH descriptor: [Opiate Alkaloids] this term only

#47 MeSH descriptor: [Morphinans] this term only

#48 MeSH descriptor: [Cocaine] this term only

#49 MeSH descriptor: [Cocaine Smoking] this term only

#50 MeSH descriptor: [Marijuana Smoking] this term only

#51 MeSH descriptor: [Hypnotics and Sedatives] this term only

#52 MeSH descriptor: [Barbiturates] this term only

#53 MeSH descriptor: [Benzodiazepines] this term only

#54 MeSH descriptor: [Heroin] this term only

#55 MeSH descriptor: [Cannabis] this term only

#56 MeSH descriptor: [Solvents] this term only

#57 MeSH descriptor: [Psychotropic Drugs] this term only

#58 MeSH descriptor: [Hallucinogens] this term only

#59 MeSH descriptor: [Tranquilizing Agents] this term only

#60 MeSH descriptor: [Nonprescription Drugs] this term only

#61 MeSH descriptor: [Behind-the-Counter Drugs] this term only

#62 MeSH descriptor: [Steroids] this term only

#63 MeSH descriptor: [Cannabinoids] this term only

#64 MeSH descriptor: [Narcotic Antagonists] this term only

#65 MeSH descriptor: [Anesthetics] this term only

#66 MeSH descriptor: [Controlled Substances] this term only

#67 MeSH descriptor: [Synthetic Drugs] this term only

#68 MeSH descriptor: [Designer Drugs] this term only

#69 MeSH descriptor: [Dopamine Agents] this term only

#70 MeSH descriptor: [Amphetamines] this term only

#71 MeSH descriptor: [Amphetamine] this term only

#72 MeSH descriptor: [Methamphetamine] this term only

#73 MeSH descriptor: [Adrenergic Agents] this term only

#74 (substance adj (use* or abuse* or misuse* or depend*)) (Word variations have been searched)

#75 (substance adj2 (disorder* or problem*)) (Word variations have been searched)

#76 (drug adj3 (use* or abuse* or misuse* or depend* or addict*)) (Word variations have been searched)

#77 ("drug use disorder*") (Word variations have been searched)

#78 ("recent drug use") (Word variations have been searched)

#79 ("prescription drug*") (Word variations have been searched)

#80 (prescription drug adj (use* or abuse* or misuse* or depend* or addict*)) (Word variations have been searched)

#81 ("nonprescription drug*") (Word variations have been searched)

#82 (nonprescription drug adj (use* or abuse* or misuse* or depend* or addict*)) (Word variations have been searched)

#83 ("non-prescription drug*") (Word variations have been searched)

#84 (non-prescription drug adj3 (use* or abuse* or misuse* or depend* or addict*)) (Word variations have been searched)

#85 ("illicit drug*") (Word variations have been searched)

#86 (illicit drug adj (use* or abuse* or misuse* or depend* or addict*)) (Word variations have been searched)

#87 (injection drug adj (use* or abuse* or misuse* or depend* or addict*)) (Word variations have been searched)

#88 (injecting drug adj (use* or abuse* or misuse* or depend* or addict*)) (Word variations have been searched)

#89 ("intravenous drug*") (Word variations have been searched)

#90 ("intravenous drug use") (Word variations have been searched)

#91 (people adj3 drugs) (Word variations have been searched)

#92 (substance adj3 patients) (Word variations have been searched)

#93 ("addictive substance*") (Word variations have been searched)

#94 ("psychotropic drug*") (Word variations have been searched)

#95 ("psychoactive drug*") (Word variations have been searched)

#96 (polydrug adj (use* or abuse* or depend* or addict*)) (Word variations have been searched)

#97 (polysubstance adj (use* or abuse* or depend* or addict*)) (Word variations have been searched)

#98 ("cannabinoids") (Word variations have been searched)

#99 ("cannabis") (Word variations have been searched)

#100 (cannabis adj2 (disorder or use* or abuse* or misuse* or depend* or addict*)) (Word variations have been searched)

#101 ("marijuana") (Word variations have been searched)

#102 (marijuana adj2 (disorder or use* or abuse* or misuse* or depend* or addict*)) (Word variations have been searched)

#103 ("narcotics") (Word variations have been searched)

#104 (narcotic adj (use* or abuse* or misuse* or depend* or addict*)) (Word variations have been searched)

#105 ("opiate*") (Word variations have been searched)

#106 (opiate adj2 (disorder or use* or abuse* or misuse* or depend* or addict*)) (Word variations have been searched)

#107 ("opioid*") (Word variations have been searched)

#108 (opioid adj2 (disorder or use* or abuse* or misuse* or depend* or addict*)) (Word variations have been searched)

#109 ("opioid dependent people") (Word variations have been searched)

#110 ("heroin") (Word variations have been searched)

#111 (heroin adj2 (disorder or use* or abuse* or misuse* or depend* or addict*)) (Word variations have been searched)

#112 ("cocaine") (Word variations have been searched)

#113 (cocaine adj2 (disorder or use* or abuse* or misuse* or depend* or addict*)) (Word variations have been searched)

#114 ("crack cocaine") (Word variations have been searched)

#115 ("solvent*") (Word variations have been searched)

#116 (solvent adj (use* or abuse*)) (Word variations have been searched)

#117 ("inhalant*") (Word variations have been searched)

#118 (inhalant adj (use* or abuse*)) (Word variations have been searched)

#119 ("benzodiazepine*") (Word variations have been searched)

#120 (benzodiazepine adj (use* or abuse* or misuse* or depend* or addict*)) (Word variations have been searched)

#121 ("hypnotics") (Word variations have been searched)

#122 ("hallucinogens") (Word variations have been searched)

#123 ("sedative*") (Word variations have been searched)

#124 (sedative adj (use* or abuse* or misuse* or depend* or addict*)) (Word variations have been searched)

#125 ("barbiturate*") (Word variations have been searched)

#126 (barbiturate adj (use* or abuse* or misuse* or depend* or addict*)) (Word variations have been searched)

#127 ("steroids") (Word variations have been searched)

#128 (steroid adj (use* or abuse* or misuse* or depend* or addict*)) (Word variations have been searched)

#129 ("non-medical use of drugs") (Word variations have been searched)

(amphetamine* adj (use* or abuse* or misuse* or depend* or addict*)).mp.

(methamphetamine* adj (use* or abuse* or misuse* or depend* or addict*)).mp.

#130 #1 or #2 or #3 or #4 or #5 or #6 or #7 or #8 or #9 or #10 or #11 or #12 or #13 or #14 or #15 or #16 or #17 or #18 or #19 or #20 or #21 or #22

#131 #23 or #24 or #25 or #26 or #27 or #28 or #29 #30 or #31 or #32 or #33 or #34 or #35 or #36 or #37 or #38 or #39 or #40 or #41 or #42 or #43 or #44 or #45 or #46 or #47 or #48 or #49 or #50 or #51 or #52 or #53 or #54 or #55 or #56 or #57 or #58 or #59 or #60 or #61 or #62 or #63 or #64 or #65 or #66 or #67 or #68 or #69 or #70 or #71 or #72 or #73 or #74 or #75 or #76 or #77 or #78 or #79 or #80 or #81 or #82 or #83 or #84 or #85 or #86 or #87 or #88 or #89 or #90 or #91 or #92 or #93 or #94 or #95 or #96 or #97 or #98 or #99 or #100 or #101 or #102 or #103 or #104 or #105 or #106 or #107 or #108 or #109 or #110 or #111 or #112 or #113 or #114 or #115 or #116 or #117 or #118 or #119 or #120 or #121 or #122 or #123 or #124 or #125 or #126 or #127 or #128 or #129

#132 #130 and #131

#133 #130 and #131 with Cochrane Library publication date between Jan 2000 and Dec 2020
